# Thiol Redox Regulation of Plant β-Carbonic Anhydrase

**DOI:** 10.3390/biom10081125

**Published:** 2020-07-30

**Authors:** Anna Dreyer, Alexander Schackmann, Alexandre Kriznik, Kamel Chibani, Corinna Wesemann, Lara Vogelsang, André Beyer, Karl-Josef Dietz

**Affiliations:** 1 Department of Biochemistry and Physiology of Plants, Faculty of Biology, University of Bielefeld, 33615 Bielefeld, Germany; anna.dreyer@uni-bielefeld.de (A.D.); Alexander.Schackmann@web.de (A.S.); kamel.chibani@uni-bielefeld.de (K.C.); c.wesemann91@gmx.de (C.W.); lara.vogelsang@gmx.de (L.V.); 2 CNRS, INSERM, IBSLor, Biophysics and Structural Biology, Université de Lorraine, F-5400 Nancy, France; alexandre.kriznik@univ-lorraine.fr; 3 Physics of Supramolecular Systems and Surfaces, Faculty of Physics, Bielefeld University, 33615 Bielefeld, Germany; andre.beyer@physik.uni-bielefeld.de

**Keywords:** *Arabidopsis thaliana*, carbonic anhydrase, site-directed mutagenesis, thiol redox regulation, thioredoxin

## Abstract

β-carbonic anhydrases (βCA) accelerate the equilibrium formation between CO_2_ and carbonate. Two plant βCA isoforms are targeted to the chloroplast and represent abundant proteins in the range of >1% of chloroplast protein. While their function in gas exchange and photosynthesis is well-characterized in carbon concentrating mechanisms of cyanobacteria and plants with C4-photosynthesis, their function in plants with C3-photosynthesis is less clear. The presence of conserved and surface-exposed cysteinyl residues in the βCA-structure urged to the question whether βCA is subject to redox regulation. Activity measurements revealed reductive activation of βCA1, whereas oxidized βCA1 was inactive. Mutation of cysteinyl residues decreased βCA1 activity, in particular C280S, C167S, C230S, and C257S. High concentrations of dithiothreitol or low amounts of reduced thioredoxins (TRXs) activated oxidized βCA1. TRX-y1 and TRX-y2 most efficiently activated βCA1, followed by TRX-f1 and f2 and NADPH-dependent TRX reductase C (NTRC). High light irradiation did not enhance βCA activity in wildtype Arabidopsis, but surprisingly in *βca1* knockout plants, indicating light-dependent regulation. The results assign a role of βCA within the thiol redox regulatory network of the chloroplast.

## 1. Introduction

The atmospheric CO_2_ concentration in the preindustrial era was close to 280 ppm. Since the beginning of industrialization in the 18th century, the CO_2_ concentration is exponentially increasing and has reached 417 ppm in May 2020 (Mouna Loa, update 5th of June 2020). Despite this increase, the current CO_2_ concentration is still limiting for C3 photosynthesis where the lyase ribulose-1,5-bisphosphate carboxylase oxygenase (RubisCO) carboxylates ribose-1,5-bisphosphate. This reaction is limited by the low K_M_(CO_2_) of RubisCO in the range of 10–12 µM, and the competing reaction of RuBP oxygenation with a K_M_(O_2_) of about 600 µM [1]. Leaf CO_2_ fixation rates of C3-plants tentatively increase threefold, if the ambient CO_2_ concentration increases to saturation [2].

The CO_2_ limitation of photosynthetic CO_2_ assimilation and plant yield formation was the driving force for evolution of carbon concentrating mechanisms (CCM). Cyanobacteria express carboxysomes as a bacterial microcompartment in order to increase the CO_2_ concentration at the active site of RubisCO [3]. Aggregates of RubisCO and carbonic anhydrase (CA) are surrounded by a proteinaceous shell permeable for HCO_3_^−^, but impermeable for CO_2_. CA generates a high CO_2_ concentration in the carboxysome. Shell breakage may cause carboxysome loss in cyanobacteria [4].

C4 plants like maize employ a CO_2_ concentration mechanism where HCO_3_^−^ is fixed by phospho-*enol*-pyruvate carboxylase (PEPC) in the mesophyll to produce oxaloacetate, which is reduced to malic acid with NADH + H^+^ as an electron donor. β-carbonic anhydrase (βCA) catalyzes the hydration of CO_2_ to HCO_3_^−^, which serves as substrate for PEPC. After transport of suitable metabolites like malic acid to the bundle sheath, CO_2_ is released in the bundle sheath by decarboxylation. Mathematical modeling of C4 photosynthesis reveals that βCA activity influences the leakiness of CO_2_ from bundle sheath cells. Sufficient βCA activity assures a low leakiness of CO_2_ out of the bundle sheath. This ensures the needed CO_2_ saturation of the Calvin–Benson cycle (CBC) [5].

CAs are metalloenzymes catalyzing the mutual conversion of CO_2_ + H_2_O ↔ H^+^ + HCO_3_^−^ with turnover numbers of >10^6^ s^−1^. The high turnover number reveals a diffusion-limited CA activity. CAs are grouped into eight classes namely α, β, γ, δ, ζ, η, θ, and ι [6]. Plant CA isoforms of the α-, β-, and γ-classes are targeted to the chloroplast, mitochondrion, plasma membrane or remain in the cytosol. The genome of *Arabidopsis thaliana* codes for 19 CA genes, namely 8 αCAs, 6 βCAs, 3 γCAs, and 2 γ-like CAs [7].

βCAs display a catalytic Zn-ion coordinated by two Cys and one His residue [8]. βCAs locate to the cytosol, plasma membrane, and mitochondrion and two isoforms localize in the chloroplast, βCA1.1 (At3g01500) and βCA5 (At4g33580) [9]. *βca1* knockout Arabidopsis plants display lower survival rates during germination and seedling growth. Growth in elevated CO_2_ rescues the wildtype phenotype [10]. Adult plants were indistinguishable from wildtype plants. A double mutant *βca1-βca4* lost its ability to close its stomata in response to high ambient CO_2_ [11]. This effect of HCO_3_^−^ on stomatal aperture could be traced to the activation of the slow anion channel associated 1 (SLAC1) via the protein kinase open stomata 1 (OST1) [12].

βCAs were frequently recovered in redox proteomics approaches. Thus, Balmer et al. [13] pulled out βCA by thioredoxin (TRX)-f and TRX-m affinity chromatography. This method uses Cys → Ser-mutated TRX variants to covalently trap TRX protein targets on TRX-bound sepharose. Trapped polypeptides are eluted under reducing conditions and identified by mass spectrometry [13]. Likewise, a similar approach with the glutaredoxin (GRX) variant GRXC4 Cys → Ser recovered βCA as well [14]. Furthermore, Marchand et al. [15] used reduced TRX-h3 as electron donor to target proteins from extracts, labeled the newly formed free thiols and identified βCA1 as one of the target proteins using MALDI-TOF. In addition, Kikutani et al. [16] showed the redox regulation of carbonic anhydrase via TRX from *Phaeodactylum tricornutum*. These results indicate that also *Arabidopsis thaliana* βCA contain redox-sensitive Cys thiols. Thiol redox regulation is a prominent mechanism to control chloroplast metabolism, in particular, the activity of the CBC. A profound understanding of the redox regulatory network has emerged. It consists of TRX and GRX as redox transmitters, many polypeptides as regulated redox targets, reactive species as final electron acceptors, and thiol peroxidases as redox sensors [17,18].

For this reason, this work was designed to address the open question whether chloroplast βCA is subject to thiol redox regulation. Following establishment of the inhibition of βCA1 from *Arabidopsis thaliana* by oxidation, we sought for the chloroplast reductant able to activate oxidized βCA. In this work, we tested the impacts of typical thioredoxins (f1, f2, m1 m2, m3, m4, x, y1, y2) or atypical multidomain thioredoxin (CDSP32) or the atypical thioredoxin reductase (NTRC) on the βCA1 activity and conformation (oligomeric state). In addition, site-directed mutation of exposed Cys residues helped identifying involved thiols. Finally, to investigate the role of βCA1 in planta, activity was determined in leaf extracts from wildtype (WT) and *βca1* knock-out mutants exposed to different light intensities.

## 2. Materials and Methods

### 2.1. Cloning

βCA1 was cloned into pET28a using the primer as depicted in Table S1. Cysteine substitutions in βCA1 were achieved by using the aqua cloning technique [19] with specific primer (Table S1). Constructs of TRX-f1, TRX-m1, TRX-m2, TRX-m4, TRX-x, TRX-y1, and TRX-y2 and chloroplastic drought-induced stress protein of 32 kDa (CDSP32) were cloned in the pET15b vector, TRX-f2 in pEXP5N TOPO, TRX-m3 in a modified version of TOPO, and NADP-dependent thioredoxin reductase C (NTRC) in pET28a. Correctness of all constructs was confirmed by DNA sequencing before use in these experiments.

### 2.2. Heterologous Expression and Purification

*E. coli* NiCo21 (DE3) cells were transformed with plasmid DNA. After induction with 100 µM isopropyl-β-D-thiogalactopyranoside, recombinant protein expression was carried out for 4 h at 37 °C and 170 rpm. Cells were harvested by centrifugation (5000× *g*, 10 min, 4 °C). Pelleted cells were resuspended in lysis buffer (50 mM NaH_2_PO_4_, 250 mM NaCl, 10 mM imidazole pH 8), disrupted by sonication (HF-Generator GM 2070 in combination with an ultrasonic converter UW2070, standard horn SH 70G, and microtip MS73, Bandelin, Berlin, Germany) and centrifuged at 12,000× *g* and 4 °C for 60 min. After passage through a chitin column (New England BioLabs, Frankfurt, Germany), the lysate was incubated with Roti ^®^ Garose-His/Ni NTA-beads (Carl Roth, Karlsruhe, Germany) under gentle shaking at 4 °C for 60 min. After exhaustive washing with washing buffer I (50 mM NaH_2_PO_4_, 250 mM NaCl, 20 mM imidazole pH 8) and washing buffer II (50 mM NaH_2_PO_4_, 250 mM NaCl, 40 mM imidazole pH 8), the his-tagged protein was eluted using the elution buffer (50 mM NaH_2_PO_4_, 250 mM NaCl, 250 mM imidazole pH 8). The eluted proteins were dialyzed over night at 4 °C against 40 mM K-phosphate pH 7.2. βCA1 and the Cys variants were dialyzed over night at 4 °C against 50 mM Na-phosphate pH 8. Prior to usage, the proteins were centrifuged for 5 min at 13,000 rpm to remove denatured protein. Protein concentrations were determined spectrophotometrically using the protein-specific molar extinction coefficient at 280 nm calculated with the Expasy ProtParam tool [20].

### 2.3. Circular Dichroism Spectra

Circular dichroism (CD) spectra in the far-UV range were recorded in a Chirascan-plus spectropolarimeter equipped with a Peltier system (Applied Photophysics Ltd., Surrey, UK). CD spectra were obtained with 50 µM βCA1 in a flat quartz cell (path length 0.01 cm) at 273 K using a scan rate of 0.5 nm/s, in the far-UV range (180 to 260 nm), and are the average of three successive measurements. The spectrum of a buffer blank was subtracted from the raw CD data, and the result was expressed as molar ellipticity per amino acid (deg cm^2^ dmol^−1^ per residue).

### 2.4. Dynamic Light Scattering

βCA1 size was determined by photon correlation spectroscopy on a dynamic light scattering (DLS) Zetasizer Nano-S model (Malvern Instruments, Malvern, UK). Purified βCA1 was analyzed by DLS at the final concentration of 320 µM either after addition of 20 mM DTT or in the presence of 500 µM H_2_O_2_. The reference buffer was Na-phosphate (50 mM, pH 8). Prior to analysis, all samples were centrifuged at 21,130× *g* at 4 °C for 30 min to eliminate disturbing particles with a hydrodynamic diameter greater than 1 µm that could interfere with the measure. A 15 µL supernatant volume was transferred into a quartz cuvette of 15 µL and the particles size measured in triplicate at 25 °C with a light diffusion angle of 173°. The data were collected in automatic mode and analyzed using the associated software DTS v4.2. Data were expressed either in intensity or after mathematical treatment using Mie theory [21] in volume percentage.

### 2.5. Size Exclusion Chromatography

Size exclusion chromatography (SEC) was done using the HiPrep 16/60 Sephacryl S200 HR column (GE Healthcare, Chicago, IL, USA). Protein thiols were reduced with 50 mM DTT at room temperature for 30 min. A PD10 column was used to desalt the protein. Part of the protein was loaded on the SEC column. The other part was oxidized with 100 µM H_2_O_2_ for 30 min at room temperature. One part of the oxidized protein was loaded on the SEC column or incubated with reduced TRX-f1 for 30 min at room temperature and then loaded on the SEC column.

### 2.6. Activity Measurements

Activity of βCA1 was measured according to [22,23]. Recombinant His_6_-tagged βCA1 was pretreated with indicated DTT concentrations for 30 min at room temperature. For the reoxidizing experiment, reduced βCA1 was desalted using Zeba Spin Desalting Columns (Thermo Fisher Scientific, Waltham, MA, USA) prior to oxidation with indicated substances. The βCA1 protein was added to 2 mL 20 mM Tris-HCl (pH 8.5 at 5 °C) with 15 µg/mL phenolred (T127.2, Carl Roth, Karlsruhe, Germany) at a final concentration of 50 nM βCA1. Then, 1.33 mL of ice-cold CO_2_-saturated water with 15 µg/mL phenolred was added to the cuvette to start the reaction at 2 °C. The reaction was monitored spectrophotometrically at 558 nm as decrease of absorbance (Cary 3500 UV-Vis, Agilent, Santa Clara, CA, USA). The Wilbur-Anderson Unit (WAU) [22,24] was determined as the time span required for the pH value to drop from 8.3 to 6.3. To determine the enzyme activity, ex vivo proteins were extracted from leaves. Pulverized plant material was mixed with 20 mM Tris-HCl (pH 8.5 at 5 °C), centrifuged at 13,000× *g* at 4 °C for 5 min, and protein content of the supernatant was determined using the Bradford assay [25]. Defined amounts of protein were subjected to the βCA1 activity measurement without prior reduction with DTT.

### 2.7. Quantification of Free Thiols

Ellman’s reagent was used to quantify the free thiols of βCA1 [26]. Absorption was read in a microplate using 160 µL assay buffer (50 mM NaPi buffer pH 8.0, 6 mM EDTA), 20 µL 6 mM 5,5′-dithiobis-(2-nitrobenzoic acid) (DTNB) dissolved in assay buffer and 20 µL sample. Data were calibrated using glutathione. After 5 min incubation at room temperature, absorption was read at 412 nm.

### 2.8. Plant Growth and Stress Application

*A. thaliana* Col_0 and *βca1* knock out lines (SALK_106570C and SALK_131447) were obtained from the Salk Institute for Biological Studies [27] and grown on soil in 10 h light (80 µmol photons m^−2^ s^−1^) at 21 °C and 14 h dark at 19 °C with 50% relative humidity. Leaves of 5-week-old plants were cut and placed on a Petri dish on ice in darkness or high light (HL: 800 µmol photons m^−2^ s^−1^), respectively. Control leaves were stressed just with HL for 1 h. After indicated time points, samples were taken and immediately frozen in liquid nitrogen. To combine cold stress with HL, the pots were placed in ice-water in HL for 1 h. Afterwards, the plants were harvested and immediately frozen in liquid nitrogen.

### 2.9. Redox Titration

Redox titration was done as previously described by [28]. Ratios of reduced and oxidized DTT at a total concentration of 50 mM in 20 mM Tris-HCl, pH 8, were used to adjust the redox potential. The potential was calculated with the Nernst equation based on an E_m_ value of −366 mV for DTT at pH 8.0 [29]. After incubation of βCA1 in the redox buffer at room temperature for 30 min, samples were mixed with loading buffer (625 mM Tris-HCl pH 6.8, 10% (*v*/*v*) glycerol, 4% (*w*/*v*) sodiumdodecylsulfate (SDS), 0.01% (*w*/*v*) bromophenol blue) and loaded on a non-reductive SDS-PAGE (12%). Afterwards, the gel was blotted to nitrocellulose. Proteins were detected using specific anti-βCA1 antibody, peroxidase-labeled secondary antibody against rabbit, ECL Substrate (GE Healthcare, Chicago, IL, USA), and X-ray films.

### 2.10. Determination of βCA1 Redox State Ex Vivo

Pulverized plant material was vortexed with 5 mM mPEG-Mal in 40 mM Tris-HCl pH 7.2 and incubated for 30 min in dark at room temperature. The samples were centrifuged at 20,000× *g* for 5 min at room temperature. Protein content of the supernatant was determined according to [25]. Then, 5 µg protein were separated by reducing SDS-PAGE (12%), transferred onto nitrocellulose membrane, and detected using specific anti-βCA1 antibody, peroxidase-labeled second antibody against rabbit, ECL Substrate (GE Healthcare, Chicago, IL, USA), and X-ray films.

### 2.11. Structural Modeling of βCA1

βCA1 structure was obtained from SWISS-MODEL [30] based on the structure of *Pisum sativum* βCA (pdb: 1ekj). From the octamer, only a dimeric subunit was used for further analysis with PyMOL version 2.4.0 [31].

### 2.12. Cysteine Conservation

In silico analyses for Cys conservation were performed with the publicly available pipeline ConCysFind (https://bibiserv.cebitec.uni-bielefeld.de/concysfind). ConCysFind evaluates the likelihood of functional conservation of post-translational modification-relevant amino acids by employing a comparative phylogeny-based approach featuring 21 plant proteomes. For every species, the most similar protein sequence to the input query containing the Cys serves as basis for the calculation of a Cys-Score, which indicates the degree of conservation of a particular Cys at a relative position over all 21 species. TSV input function and UniProt ID of *A. thaliana* βCA1 (P27140) were used for submission of the survey. Analysis was performed following ConCysFind-default settings (bibiserv.cebitec.uni-bielefeld.de/concysfind?id=concysfind_manual). A particular Cys was considered conserved when featuring a Cys-Score > 0.5 and a corresponding *p*-value < 0.5.

## 3. Results

### 3.1. Redox-Dependent Activity of βCA1

**Figure 1:**
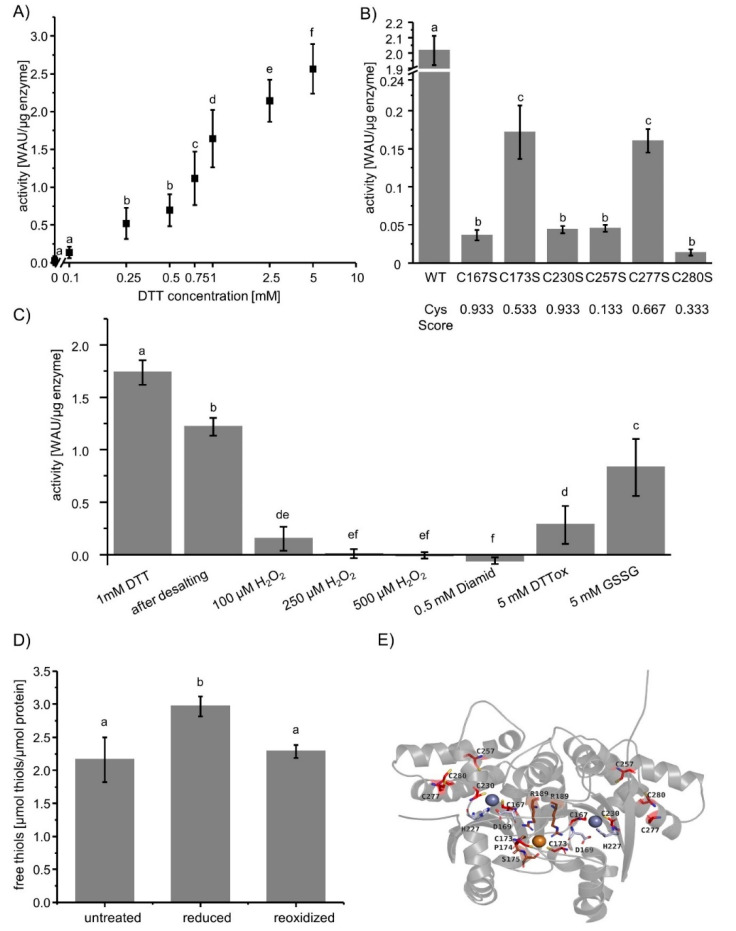
Redox-dependent activity of wildtype βCA1 and its Cys → Ser variants. (**A**) βCA1 activity after treatment with increasing DTT concentrations for 30 min at room temperature. Letters a-f indicate significant differences at *p* ≤ 0.05, as calculated using one-way ANOVA with post hoc Tukey HSD test. Data are means ± SD of *n* ≥ 13 from two independent protein purifications. (**B**) Activity of βCA1 and its Cys variants after preincubation with 1 mM DTT for 30 min at room temperature. Data are means of *n* ≥ 6 ± SD from two independent protein purifications. Significant differences at *p* ≤ 0.05 were calculated using one-way ANOVA with post hoc Tukey HSD and are represented by letters a–c. Furthermore, the Cys-Score >0.5 indicates whether the Cys residues are conserved in plant βCA. (**C**) Activity of βCA1 after reduction with 1mM DTT, desalting, and re-oxidation with different amounts of H_2_O_2_, diamide, DTT_ox_, and oxidized glutathione. Data are means ± SD of *n* ≥ 6. Letters a-f indicate significance of difference calculated with one-way ANOVA and post hoc Tukey HSD. (**D**) Quantification of thiols of untreated, or reduced (1 mM DTT) and desalted βCA1 either labeled directly or after oxidation with 100 µM H_2_O_2_. Thiols were quantified using Ellman’s reagent. Data represent means ± SD of *n* ≥ 4 from two independent protein purifications. Significance of difference was calculated using one-way ANOVA and post hoc Tukey HSD and are represented by letters a and b. (**E**) Structure of the homodimer of βCA1 based on the x-ray structure of CA of *Pisum sativum* (pdb: 1ekj). Cys are marked in red, zinc binding amino acids, besides C167 and C230, are marked in gray, and copper binding amino acids, besides C173, are marked in brown. The zinc and copper ions are shown as spheres in gray and brown, respectively. The homodimer has two active sides where the zinc is bound by C167, D169, H227, and C230 in each subunit. The copper ion is bound by C173, P174, S175, R189 from Chain A and C173 and R189 from Chain B.

To address thiol redox state-dependent effects on βCA1 activity, an enzyme assay with phenol red was established to monitor the pH change due to hydration of CO_2_. βCA1 was incubated without reductant or with different concentrations of DTT prior to the activity measurement. βCA1 showed a strong redox-dependent activity (Figure 1A). Oxidized βCA1 was inactive and did not accelerate the rate of spontaneous CO_2_ hydration. Reducing conditions stimulated the βCA1 activity. Oxidation with 100 µM H_2_O_2_ resulted in a negligible activity of 0.04 ± 0.04 WAU/µg enzyme. Inhibition was also seen following reduction and subsequent treatment with 100 µM H_2_O_2_ (Figure 1C). Likewise, reductively activated βCA1 lost its activity upon treatment with 0.5 mM diamide, 5 mM DTT_ox_, or 5 mM oxidized glutathione. In contrast to βCA1 from *A. thaliana*, the αCA from *Sulfurihydrogenibium azorense*, the CA known for its high catalytic efficiency [32], remained active upon oxidation (Figure S1), which could be due to the fact that no cysteine is involved in the zinc binding [33].

In order to pinpoint to involved Cys residues we generated a Set of Cys → Ser variants (Figure 1B). Mutation of any Cys caused a significant decrease of activity. Oxidation of zinc-binding cysteines causes a loss of zinc binding capability and therefore a loss of activity [34]. Therefore, the mutation of the conserved C167 and C230, which are within the zinc binding motif, caused the expected drop of activity from 2 WAU to <0.05 WAU (Figure 1B). Interestingly, the mutation of the other Cys also caused a major loss of activity. C230, C257, C277, and C280 form a circle with distances between the free thiols of 6.7 to 9.8 Å (Figure S2). Slight moving of two nearby Cys thiols narrowed the distance to about 7Å and enabled the formation of an intramolecular disulfide bridge [35]. Inside this ring, the intramolecular disulfide can switch between the four Cys to ensure a reduced C230, which is necessary for the zinc binding and, therefore, for the activity of the protein. Quantification of thiols in untreated and reoxidized protein revealed two reduced cysteines. The two DTNB-reactive cysteines likely are C167 and C230, since oxidation of zinc-binding cysteines by low H_2_O_2_ concentrations or air oxygen is slow [34]. During reduction, a third cysteine gets reduced, this has to be C277, since it is the only other cysteine with an accessible surface area. Thus, C277 likely is the redox regulatory cysteine. Buried and hence DTNB-inaccessible C167 and C230, which are within the disulfide exchange ring, may stabilize C277 and form the disulfide bridge upon oxidation. It should be noted that the result does not unequivocally demonstrate the existence of a disulfide bond in oxidized βCA1, and therefore H_2_O_2_-induced sulfenylation of one cysteine cannot be excluded. Mutations of these cysteines should not result in inhibition of the enzyme by redox-regulation, since activity measurements were done after reduction with DTT. Therefore, the mutations themselves likely cause the loss of activity, since Cys-Ser mutations can alter the stability, structure, aggregation, and therefore, the activity of proteins [36,37].

### 3.2. Redox State-Dependent Structure and Oligomeric State of βCA1

**Figure 2:**
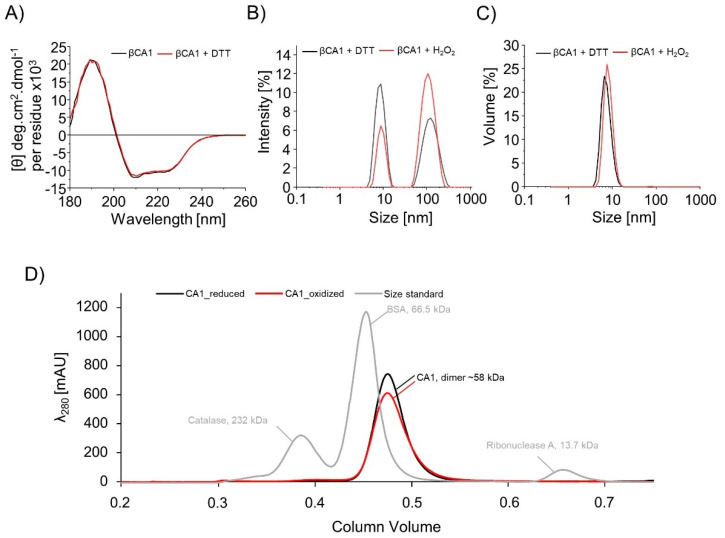
Determination of the oligomeric state of βCA1. (**A**) Superimposed far UV spectra of βCA1 with or without 10 mM DTT. (**B**) Dynamic light scattering (DLS) curves representing the βCA1 light diffusion intensity as function of the size in nm and (**C**) the βCA1 volume signal as function of the size in nm. βCA1 (320 µM) was analyzed by DLS after treatment with 20 mM DTT and 500 µM H_2_O_2_, respectively. (**D**) Size exclusion chromatography of βCA1 after treatment with 50 mM DTT and re-oxidation with 100 µM H_2_O_2_. Reduced βCA1 is shown as black line and oxidized βCA1 is shown as red line, size standard of the SEC run is shown in gray.

The impact of oxidation and reduction on the structure and oligomeric state of βCA1 was assessed in order to link possible conformational changes to activity. CD spectra were recorded from βCA1 treated with or without 10 mM DTT. The untreated purified protein is oxidized since its purification was done under non-reducing conditions. Both the oxidized and reduced form showed the same CD spectra (Figure 2A). The structure consisted of 31% α-helices, 20% β-sheets, 17% turns, and 32% random coils. Next, DLS analyses were carried out with high concentrations of βCA1, either treated with DTT or H_2_O_2_. For each sample, two peaks were detected, which corresponded to hydrodynamic diameters of around 7.5 nm and around 100 nm, respectively (Figure 2B). Analysis of the atomic structure based on the X-ray structure of the βCA of *Pisum sativum* (pdb: 1ekj) gave longitudinal dimensions of 7 nm for the dimer (Figure S3) and 12 nm for the octamer. The smaller value is in good agreement with the dimensions of the βCA1 dimer. The higher value indicates the presence of high molecular weight aggregates. Oxidation increased the amount of aggregates. However, the conversion of the sample intensity to volume by the software algorithm showed that aggregates represent only 0.2% of the analyzed sample, and the dimer essentially was the only significant population (Figure 2C). Measured hydrodynamic diameters for reduced βCA1 and oxidized βCA1 are 7.3 ± 0.2 nm and 8.1 ± 0.5 nm. Together with the SEC of reduced and re-oxidized βCA1 (Figure 2D), the results indicate that βCA1 is a homodimer with minor redox-dependent conformational changes. In the dimer–dimer interface, C173 of both subunits are in close proximity to each other (3.8 Å), which could enable the formation of an intermolecular disulfide bond to stabilize the homodimer. But non-reducing SDS-PAGE of redox titrated βCA1 excluded stoichiometric formation of dimers via C173 under oxidizing conditions (Figure S4).

### 3.3. Thioredoxin-Dependent Activity of βCA1

**Figure 3:**
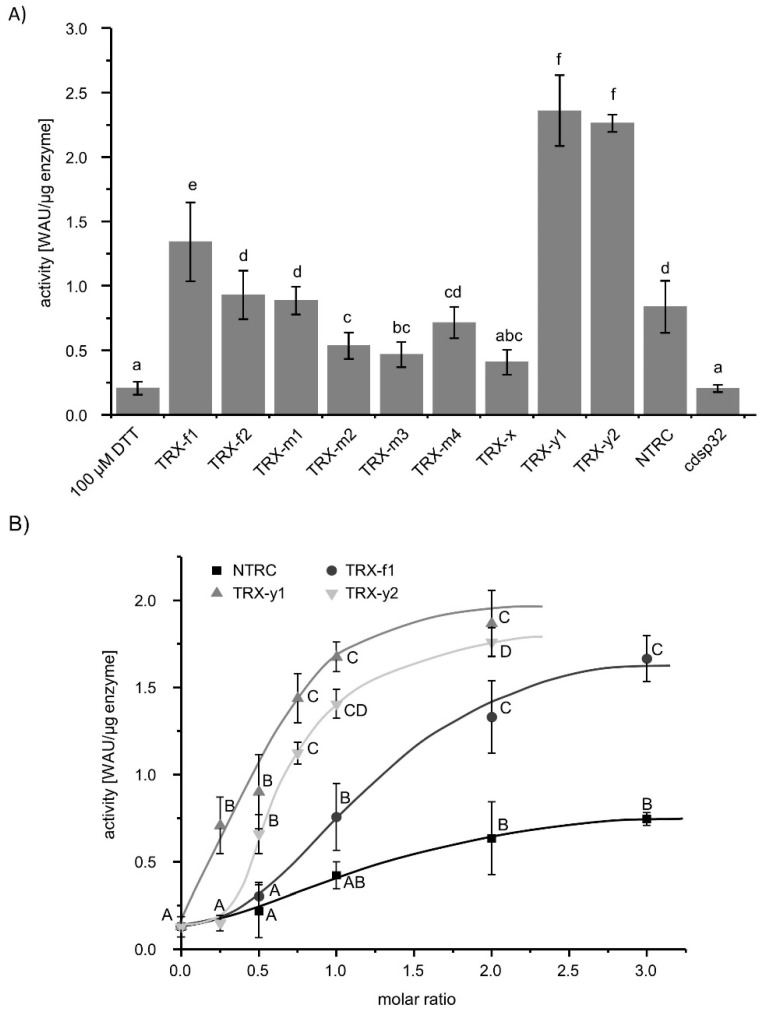
Activation of βCA1 by thioredoxin (TRX). (**A**) Activity of βCA1 after 30 min incubation with 100 µM DTT with or without equimolar amounts of different chloroplast TRXs. Data represent the mean of *n* ≥ 10 ± SD from two independent protein purifications. Letters a-g indicate significant differences of *p* ≤ 0.05 calculated with one-way ANOVA and post hoc Tukey HSD. (**B**) Concentration dependent influence of TRX-f1, TRX-y1, TRX-y2, and NTRC on the activity of βCA1. βCA1 was incubated with 100 µM DTT and the indicated molar ratio of the TRXs for 30 min and then subjected to the activity measurement. The values represent the mean of *n* ≥ 8 ± SD from two independent protein purifications. The letters A-D indicate significant differences of *p* ≤ 0.05 within each TRX-dependent activity measurement of βCA1. Calculation was done with one-way ANOVA with post hoc Tukey HSD test.

βCA1 is reported as the physical interaction partner of TRX [13,15,16]. Therefore, TRXs might function as reductant of βCA1 and increase its activity. To this end, the acceleration of acidification was determined with βCA1 preincubated with 100 µM DTT in combination with different TRXs. Afterwards, they were subjected to the βCA1 activity test. Except TRX-x and TRX-like protein CDSP32, all TRXs increased the formation rate of carbonic acid from CO_2_ by βCA1 in comparison to the control with just 100 µM DTT (Figure 3A). TRX-y1 and -y2 fully activated the βCA1 (Figure 1). CDSP32 was as ineffective as 100 µM DTT. Therefore, TRX-y1 and TRX-y2, together with TRX-f1 and NTRC, were chosen for further analysis of optimal molar ratio between βCA1 and TRX-y1, -y2, -f1, and NTRC (Figure 3B). A TRX-y1/βCA1 molar ratio of 0.25 significantly increased βCA1 activity, whereas TRX-y2 was required at a ratio of 0.5 to activate βCA1 to the same level. A threefold higher concentration of NTRC or an equimolar ratio of TRX-f1 was needed to activate βCA1 to the same level. The molar ratio of the different TRXs had a great impact on the activation and, hence, reduction capability. Thus, it appears that TRXs mediate the reduction of βCA1. Although TRX-f1 and NTRC had an impact on the activity of βCA1, they had no impact on the quaternary structure or oligomerization as shown with circular dichroism and size exclusion chromatography in Figure S5.

### 3.4. Determination of the Redox State of βCA1 Ex Vivo from High Light-Treated Cut Leaves

**Figure 4:**
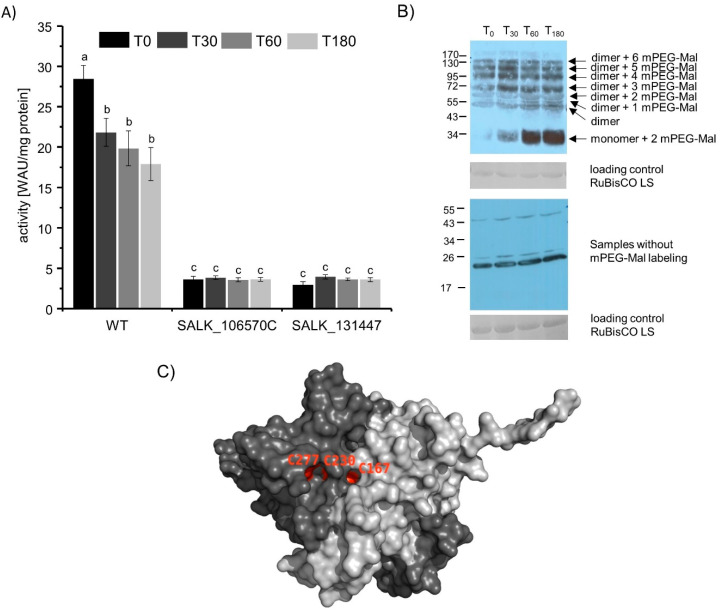
Effect of high light on CA activity and thiol redox state. Cut leaves were exposed to high light for 0, 30, 60, or 180 min. (**A**) *Ex vivo*-CA activity in whole leaf extract of WT, SALK_106570C, and SALK_131447 plants. The CA activity of the knock out lines of βCA1 ranged near 20% of WT CA activity. Data represent the mean of *n* ≥ 12 ± SE from three independent experiments. Significance of difference is indicated by letters a-c with *p* ≤ 0.05, calculated with one-way ANOVA and post hoc Tukey HSD test. (**B**) Western blot of the samples treated as in (A) with or without mPEG-Mal labeling and their corresponding loading control. Higher bands indicate additional labeling with mPEG-Mal due to addition of ~ 5kDa per accessible thiol in βCA1. (**C**) Accessible surface of βCA1 dimer (based on 1ekj). Only C167, C230, and C277 among the six Cys are accessible at the surface area of βCA1 and therefore, undergo alkylation via mPEG-Mal.

To investigate the role of βCA1 in planta, excised leaves from WT COL_0 and *βca1* knock out plants (Figure S6) were stressed for 0, 30, 60, and 180 min with high light (HL: 800 µmol photons m^−2^ s^−1^). Proteins were isolated from the HL-treated plant material and used for CA activity measurement. The differences in CA activity of WT plants in comparison to *βca1* knock out plants is remarkable. Since the only difference in the two SALK lines in comparison to the WT plants is the lack of βCA1 protein, the differences in activity reflects the βCA1 activity. βCA1 activity represents about 80% of the total CA activity (Figure 4A).

During a prolonged HL treatment, WT plants showed a decrease in total CA activity, whereas the CA activity in the *βca1* plants stayed constant. Therefore, the activity of the other plant CAs had to be constant, whereas the βCA1 activity decreased due to a possible oxidation during the HL treatment (Figure 4).

To check the redox state of βCA1, free thiols of the WT proteins were labeled with mPEG-Mal, separated by SDS-PAGE, blotted to nitrocellulose membrane, and immune labeled using specific βCA1 antibodies. Figure 4B shows increasing intensity of bands with lower molecular mass upon prolonged HL treatment. Three Cys residues are surface-exposed (Figure 4C) and may be oxidized during HL treatment. Correspondingly, CA activity decreased in HL-exposed leaf extracts.

### 3.5. β. CA1 Activity from Plants Treated with High Light or High Light Combined with Cold

**Figure 5:**
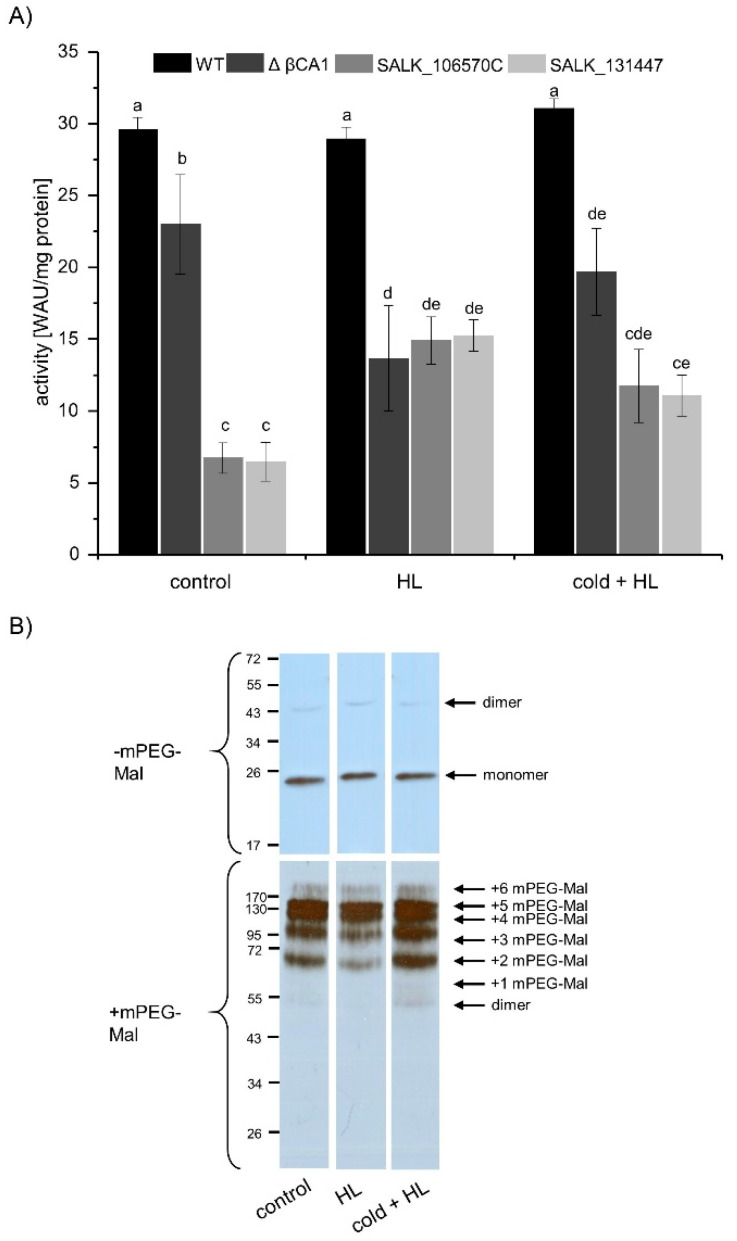
Effect of high light (HL) or combined HL and cold on CA activity. (**A**) CA activity in extracts of WT, SALK_106570C, and SALK_131447 plants treated with HL or cold together with HL for 1 h. ΔβCA1 activity was calculated as difference between the WT CA activity and the mean activity of the two *βca1* knock out lines from the corresponding treatment. Data represent the mean ± SE of *n* ≥ 8 from three independent experiments. Significant differences of *p* ≤ 0.05 are indicated by different letters a-e. Significance of difference was calculated using one-way ANOVA with post hoc Tukey HSD test. (**B**) Western blot with specific βCA1-antibody of treated WT plant material with and without mPEG-Mal labeling of free thiols.

In the next step, whole plants were stressed with HL and HL in combination with cold for 1 h. Whole leaf proteins were isolated to measure the CA activity. Besides the WT and *βca1* knock out lines, the ΔβCA1 activity was calculated as difference of the WT and the averaged SALK line activities. The ΔβCA1 activity under control conditions represented about 80% of total CA activity in *A. thaliana* (Figure 5A). The different treatments showed no effect on total CA activity, but the CA activity increased in both SALK lines equally. Therefore, the ΔβCA1 activity likely decreased during the HL and the cold + HL treatment, but the other CAs compensated the loss of activity of the βCA1 protein.

The Western blot of whole leaf proteins without mPEG-Mal and specific βCA1 antibodies showed the same expression level of βCA1 protein. But the mPEG-Mal labeled samples showed a different pattern. Especially the lower bands of the cold + HL treated plants had a higher intensity. This indicates oxidation of βCA1 during cold + HL treatment. Although the Western blot without mPEG-Mal label indicates similar βCA1 amounts under the different treatments, the Western blot with mPEG-Mal labeling indicates a lower expression of βCA1, which is fitting to the decreased ΔβCA1 activity after HL treatment. In summary, both treatments resulted in a slight oxidation of βCA1 and a decreased ΔβCA1 activity. The loss of activity likely was compensated by the other CAs in *A. thaliana*.

## 4. Discussion

The recovery of βCA in many redox proteomics studies, in particular using TRX-thiol trapping approaches [13,14,15], indicated that βCA1 may be target of redox regulation. Figure 1 shows that about 1 mM DTT is needed to activate βCA1 by about 50% under our conditions. The same activation is achieved in the presence of TRXs and 100 µM DTT. DTT at this concentration failed to reduce and activate βCA1 (Figure 3A). This result shows that βCA1 can be fully switched between an oxidized inactive and a reduced active form. This could be due to the ring of cysteines, including C230, which is part of the zinc-binding site. This ring could stabilize the redox state of C230 and, thereby, the binding of zinc by switching the disulfide between the cysteines C230, C257, C277, and C280 (Figure S2). Possibly this ring stabilizes C277. C277 is hypothesized to act as redox sensitive cysteine of the enzyme, since quantification of free thiols revealed two DTNB-accessible thiols under oxidizing conditions. C230 and C167 bind zinc and are only very slowly oxidized by air oxygen or low concentrations of H_2_O_2_ [34]. Therefore, the other free thiol under reduced conditions has to be C277, since it is the only other cysteine with an accessible surface area.

It was surprising that any Cys → Ser exchange inhibited the enzyme. Possibly, each cysteine replacement in the ring collapses the structure and the intramolecular disulfide-dithiol switch fails to activate the enzyme [36,37]. Thiol switches are commonly used throughout the kingdoms of life to adjust process activities in dependence on cellular redox state. The large families of TRXs and GRXs in plants like *A. thaliana* and *Populus trichocarpa* [38,39] underline the significance of redox regulation in the lifestyle of plants. Considering the subcellular distribution of thiol redox network components within plant cells, the plastid appears to be the organelle with the most sophisticated and complex network for thiol redox regulation.

The chloroplast contains 20 TRXs and TRX-like proteins. TRXs function as redox transmitters, transferring electrons from redox input elements like ferredoxin and NADPH to target proteins. Our data show that TRXs function as disulfide reductases of βCA1. Interestingly, the efficiency of distinct chloroplast TRXs varies profoundly (Figure 3). Recently it was shown that TRXs play a decisive role also in target protein oxidation [40]. Exemplified with enzymes of the CBC and NADPH malate dehydrogenase known to be thiol-redox regulated, these authors showed that deactivation depended on the presence of the proper TRXs and oxidized 2-cysteine peroxiredoxin (2-CysPRX) during the transition from light to darkness. This transition requires rapid inactivation of the energy-consuming CBC. The 2-CysPRX is a thiol peroxidase, which reduces reactive oxygen species by use of a catalytic thiol, the so called peroxidatic thiol. Thiol peroxidases function as redox sensors and are important in the thiol-redox regulatory network for oxidation of target protein. Only the balance between reducing and oxidizing input allows for fine-tuned adjustment of the redox state of target proteins like malate dehydrogenase, ribulose-5-phosphate kinase or fructose-1,6-bisphosphatase [17,41], and likely of βCA1. Recent interactome study of 2-CysPRX showed interaction between βCA1 and 2-CysPRXA and affected its activity [42] Activity measurements and mPEG-Mal-labeling during βCA1 extraction from stressed leaves indicate that thiol oxidation of βCA1 occurs (Figure 4 and Figure 5), but not necessarily as an on/off-switch, e.g., between light and darkness, but rather in a more subtle manner in dependence on light intensity.

The TRX type specificity of βCA1 activation was peculiar. TRX-y1 and -y2 were most effective and are known as reductants of peroxiredoxin Q (PRXQ) [43]. TRX-y1 is preferentially expressed in seeds > roots > mature siliques >> leaves, while TRX-y2 is strongly expressed in leaves [43]. PRXQ is associated with the thylakoid membrane and protects photosynthesis [44]. TRX-y1 was more than 20-fold more effective than TRX-f1 in activating βCA1 at low molar stoichiometry. TRX-y2 activated βCA1 slightly less efficient than TRX-y1 (Figure 3B). The new role of thiol peroxidases as TRX oxidases in redox regulation [40] could also be shown for PRXQ [45]. It is tempting to hypothesize that PRXQ, TRX-y, and βCA1 interact within the thiol redox regulatory network. In this scenario, the redox state of TRX-y would determine the redox state of βCA1 and the TRX-y redox state in turn would be controlled by the rate of reduction by ferredoxin-dependent TRX reductase and its oxidation by PRXQ. This could be explored in future work using *prxQ* and *trxy* knockout plants.

While these considerations in context of redox regulation appear straight forward, the function of βCA1 in chloroplasts of C3-plants and the significance of thiol switching of βCA1 remain to be explored. βCA1 catalyzes the conversion of HCO_3_^−^ to CO_2_ and may facilitate the substrate delivery to RubisCO. The contribution of βCA was simulated by mathematical modeling and ranged between 5% and 9% [46,47]. While such an increase in photosynthetic rate may justify the expression of βCA to increase competitiveness and fitness, thiol switching appears unnecessary. Other authors assign a role to βCA in pH homeostasis, chloroplast lipid metabolism, pathogen defense, and seedling survival (reviewed by [48]).

Interestingly, a certain fraction of chloroplast CA is associated with the thylakoid membrane where it interferes with photosystem II [49]. Inhibitors of CA such as acetazolamide and ethoxyzolamide also inhibit photosystem II [48]. This observation was explained by the protective effect of HCO_3_^−^ on the oxygen evolving complex [50]. HCO_3_^−^ has a positive effect on photophosphorylation, which is the ATP synthesis of thylakoids. Fedorchuk et al. [51] suggested that CA participates in proton transfer into the thylakoid lumen by increasing the local HCO_3_^−^ concentration and its transport into the lumen. Following import, CA-dependent dehydration releases H^+^ and CO_2._ The H^+^ may participate in easing the reduction of dehydroascorbate to ascorbic acid.

Dioscorins, a major tuber storage protein of Yam, functions both as CA and dehydroascorbate reductase (DHAR) [52]. The main reason these proteins are able to facilitate both functions is a His-Asp catalytic dyad. Although the three-dimensional structures of dioscorins S and αCAs are similar, the active site is not. Especially the αCAs lack Asp, which is necessary for the His-Asp catalytic dyad. Therefore, the Asp and His are not able to form a hydrogen bond, act as general base catalyst and His 227 is not able to transfer a proton from a nearby water to dehydroascorbate. Two protonation steps by His 227 would be needed for the formation of ascorbate. At the same time, the released hydroxide would react with carbon dioxide to bicarbonate [52]. But in comparison to the αCAs, the βCA1 has two Asp close to His 227 [8], namely Asp 169, which is part of the zinc-binding pocket and Asp 315. Furthermore, αCAs are zinc-binding proteins in contrast to discorin, therefore DHAR and CA activity of discorin is Zn-independent. But as described by [53] a mutation of Q114H and addition of zinc results in an increased CA activity, but decreased DHAR activity. Both observations could be an indication for a Zn-independent second function of βCA1, namely function as DHAR which should be analyzed in future studies.

Another protein usage of the H^+^ could occur in ATP synthesis and the CO_2_ evades from the thylakoids as mentioned above. Redox regulation is well-established for processes in photosynthetic electron transport, e.g., thiol switching of F-ATP synthase to control photophosphorylation [53] and cyclic electron transport via disulfide reduction in proton gradient 5-like (PGR5-like) [54]. Therefore, redox regulation of CA may be another element in redox-dependent optimization of the photosynthetic performance in plants. Time-resolved analysis of photosynthetic electron transport in wildtype and CA-deficient plants may provide insight into the role of βCA1 in photosynthesis.

## 5. Conclusions

Our study proves the existence of a thiol switch mechanism controlling chloroplast βCA1 activity. The thiol redox state of βCA1 is linked to the TRX system. This result provides an explanation for the detection of βCA1 in earlier redox proteomics analyses. Among the tested recombinant TRXs, TRX-y1 and TRX-y2 most efficiently activated oxidized inactive βCA1. Other chloroplast TRXs and TRX-like proteins like CDSP32 were inefficient or unable to activate βCA1. Mutation of any Cys residue of βCA1 partially or completely inhibited its activity. The thiol redox switch had little influence on conformation and did not affect quaternary structure. Changes in thiol redox state of βCA1 occur in vivo as detected in leaf extracts of wildtype Arabidopsis upon exposure to high light. It may be hypothesized that thiol redox regulation of CAs is needed for fine-tuned adjustment of photosynthetic processes such as photophosphorylation.

## Supplementary Materials

The following are available online at https://www.mdpi.com/2218-273X/10/8/1125/s1; Figure S1: Redox-dependent activity and structure of α-CA from *Sulfurihydrogenibium azorense*, Figure S2: Cysteine ring of the dimeric βCA1 structure, Figure S3: Molecular dimension of the βCA1, Figure S4: Western Blot of redox-titrated βCA1, Figure S5: Influence of TRX-f1 and NTRC on the conformation of βCA1, Figure S6: Characterization of βca1 knock out lines (SALK_106570C and SALK_131447), Table S1: Primers used in this study for cloning.
