# Supplementary material for: Thiol Redox Regulation of Plant β-Carbonic Anhydrase
Source: Biomolecules. 2020 Jul 30;10(8):1125. doi: 10.3390/biom10081125 (PMC7463553; doi:10.3390/biom10081125)
Supplement: Supplementary file 1 [file biomolecules-10-01125-s001.pdf]

Suppl. Fig 1

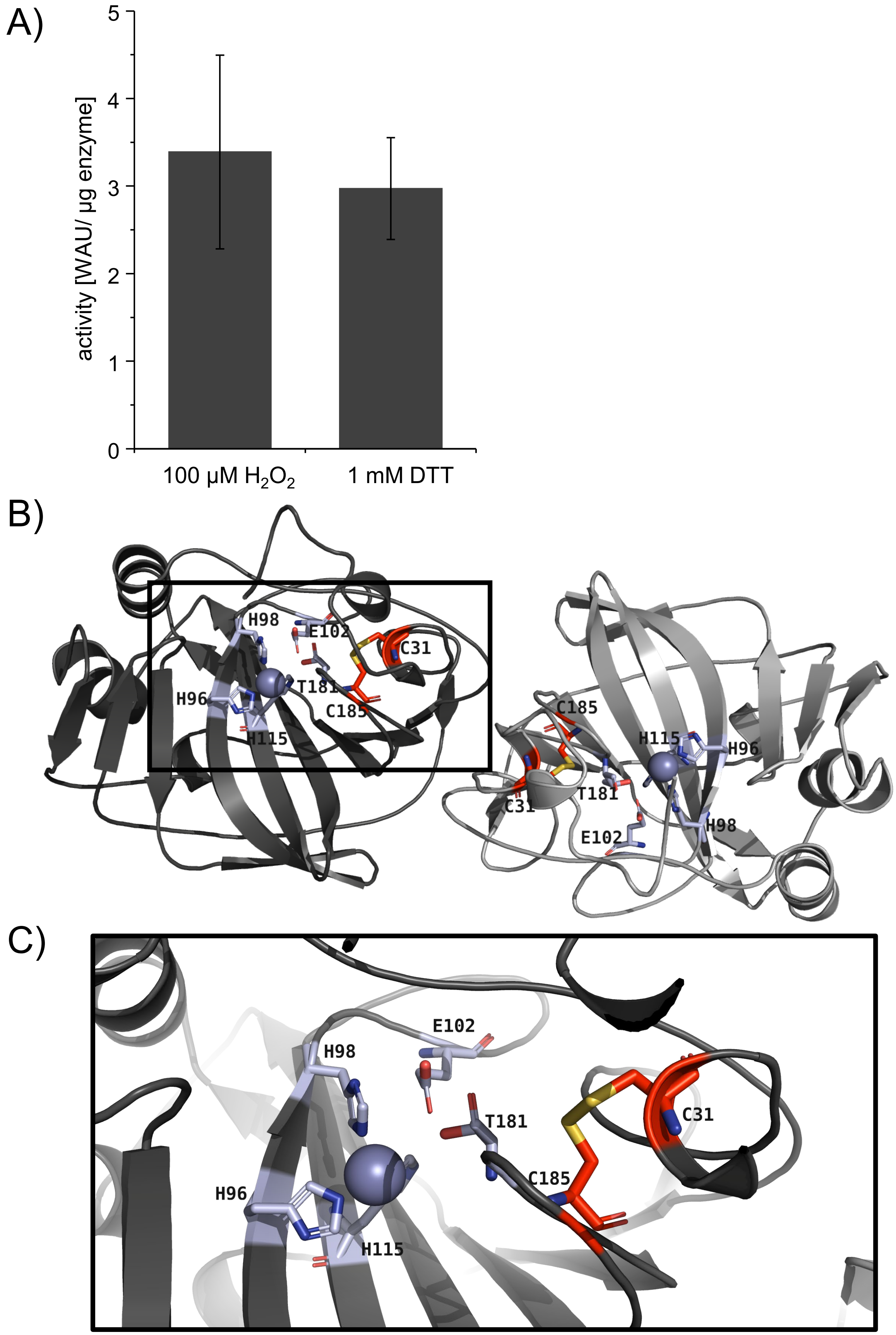

Figure S1 Redox-dependent activity and structure of α-CA from *Sulfurihydrogenibium azorense*. A) Activity of αCA after treatment with 100 µM H_2O_2 and 1 mM DTT, respectively. Data represent the mean of n = 4 ± SD. The difference between reducing and oxidizing conditions was insignificant as calculated using One-Way ANOVA with post-hoc Tukey HSD. B) Structure of αCA (pdb: 4x5s.1). C) Magnified zinc binding structure of αCA. H96, H98, E102, H115 and T181 are binding the zinc ion and are marked in light blue. C31 and C185 are marked in red.

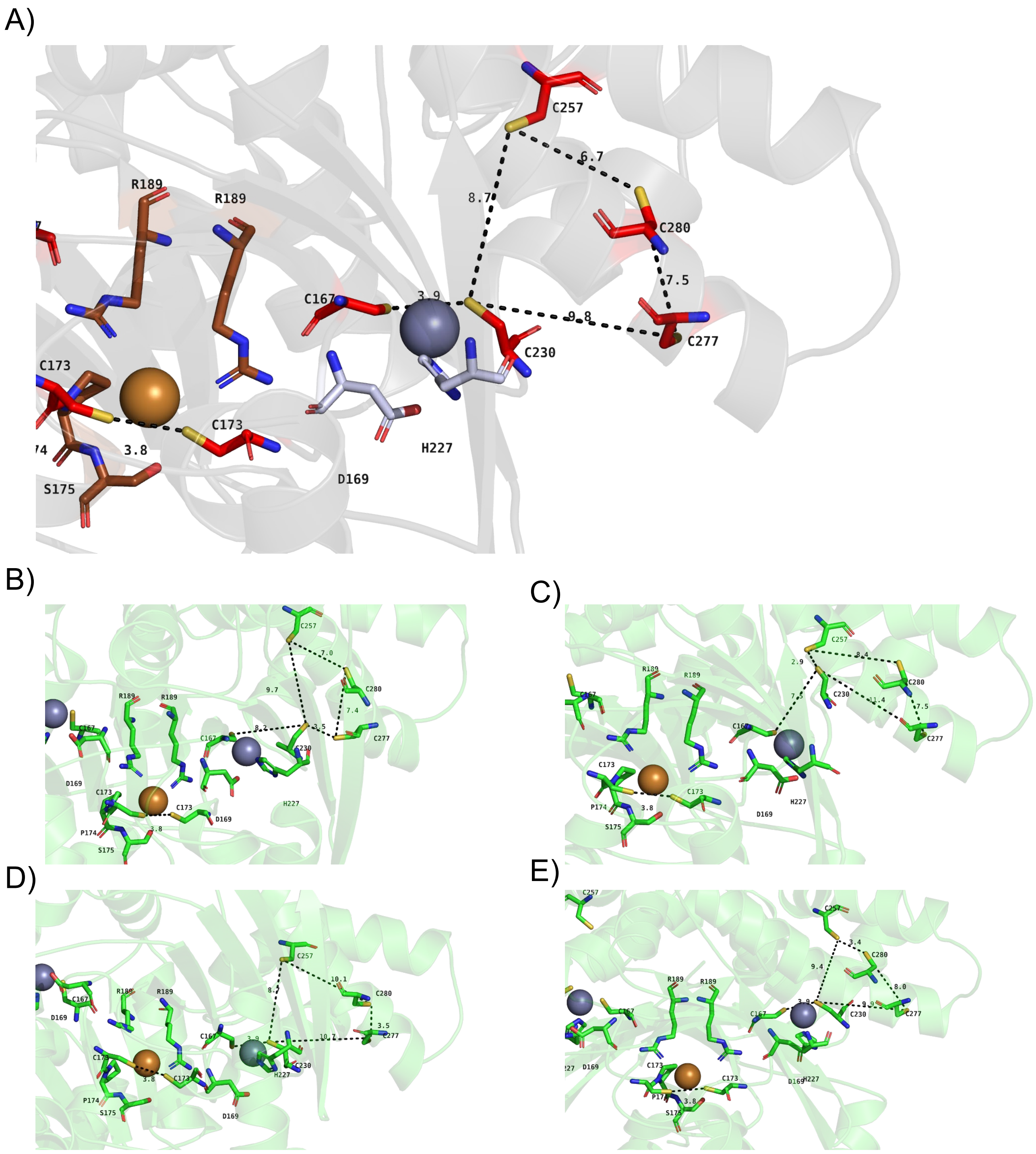

Figure S2 Cysteine ring of the dimeric βCA1 structure. A) Unmodified structure of βCA1 based on 1ekj. Cys are marked in red, the Zn-binding amino acids in gray and the copper binding amino acids in brown. The distances between the thiols of the Cys are shown in Å. B) C) D) and E) represent the structures after moving two Cys each that are close to each other.

Suppl. Fig 3

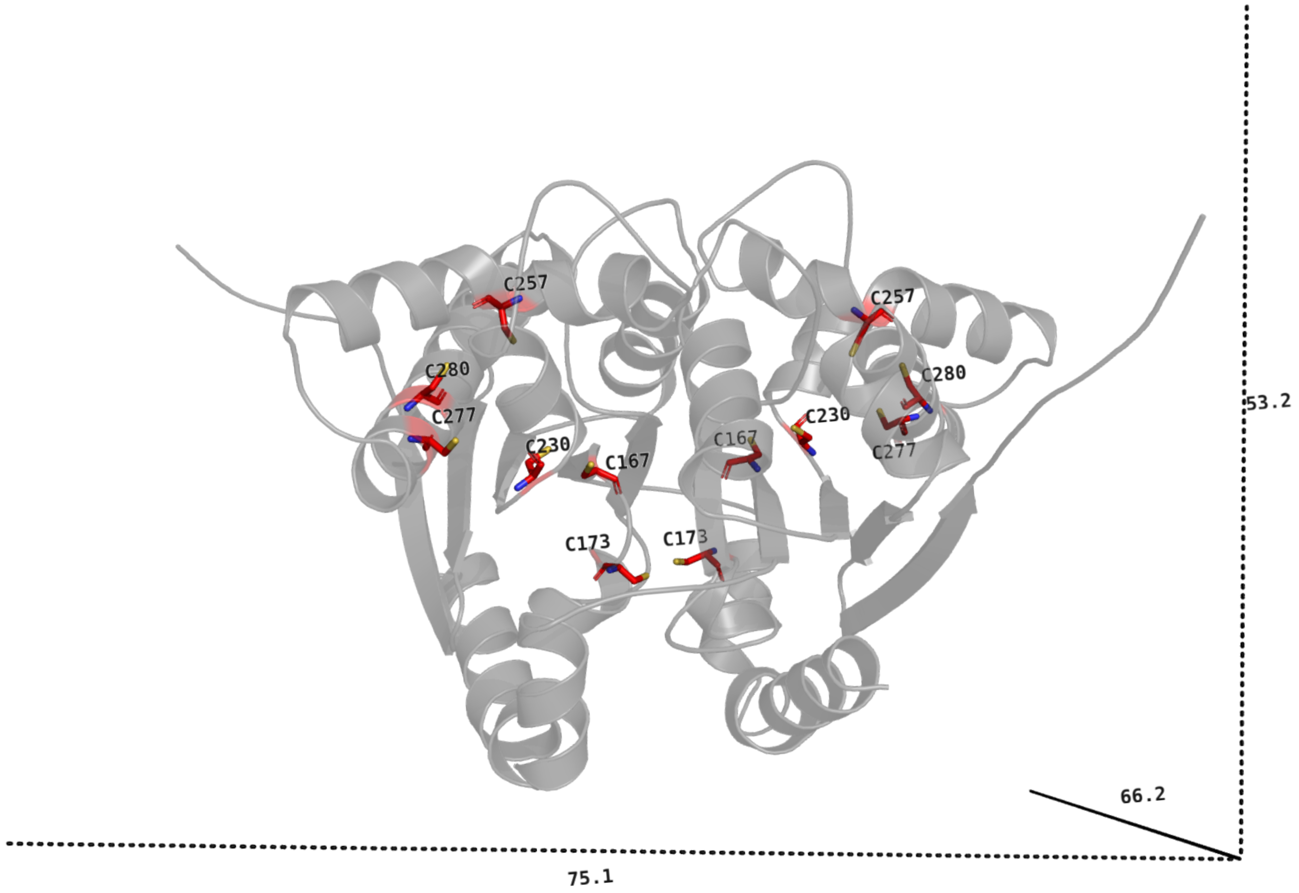

Figure S3 Molecular dimension of the βCA1. The dimensions are shown in Å and the Cys are marked in red.

Suppl. Fig 4

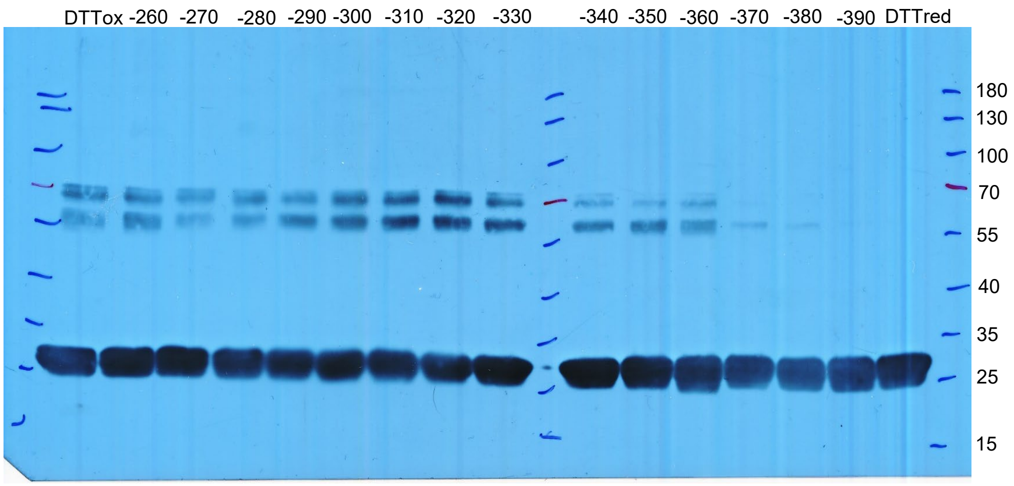

Figure S4 Western Blot of redox-titrated βCA1. After incubation with 50 mM $DTT_{red/ox}$ proteins were separated using non-reducing SDS-PAGE.

Suppl. Fig 5

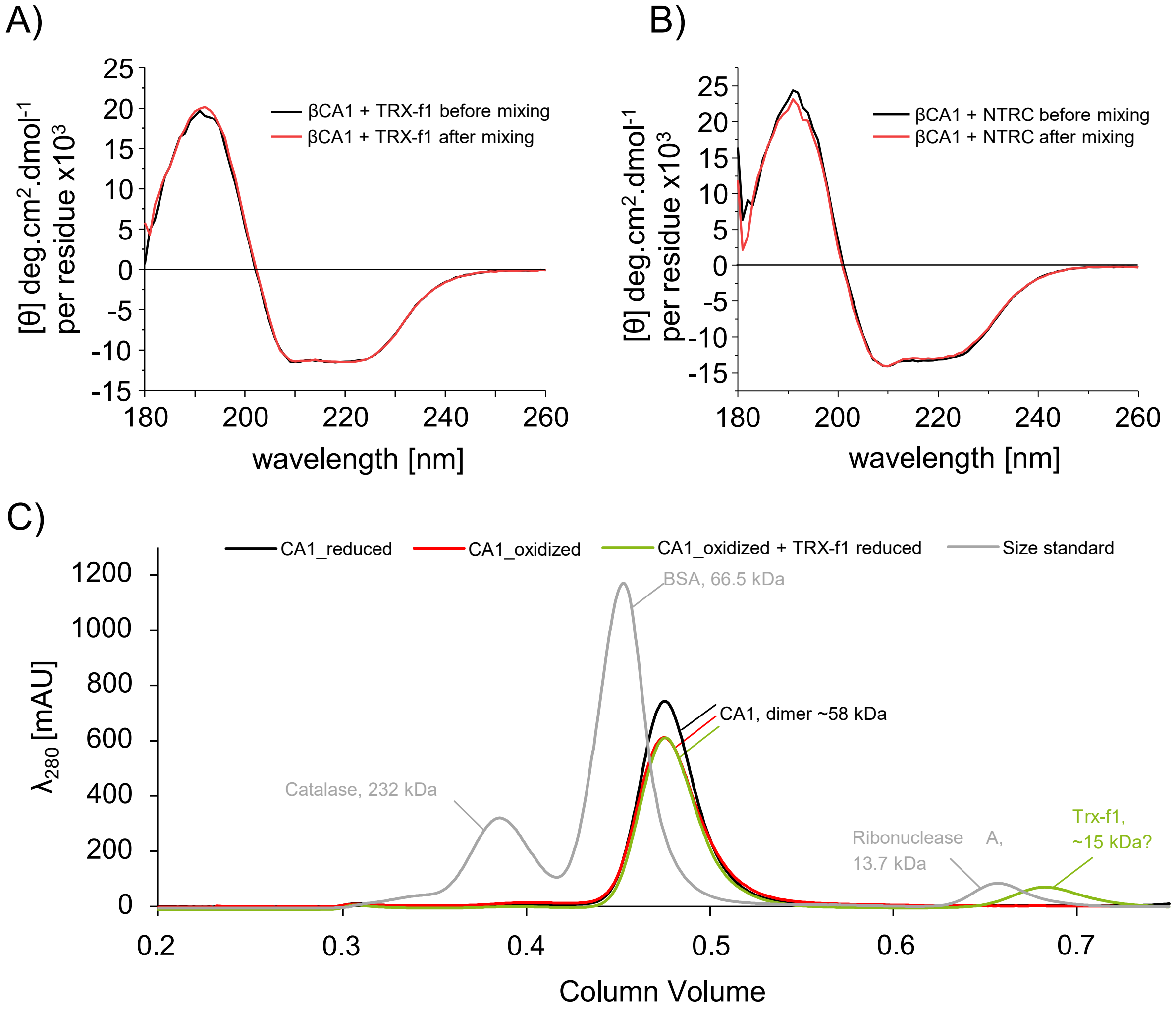

Figure S5 Influence of TRX-f1 and NTRC on the conformation of βCA1. Superimposed far UV CD spectra of βCA1 with A) TRX-f1 or B) NTRC, before mixing and after mixing of both proteins. C) Size exclusion chromatography of βCA1 after treatment with 50 mM DTT and reoxidation with 100 µM H_2O_2. After reoxidation βCA1 was incubated with reduced TRX-f1 and then subjected to SEC. Reduced βCA1 is shown as black line and oxidized βCA1 is shown as red line, oxidized βCA1 + reduced TRX-f1 is shown as green line, size standard of the SEC run is shown in gray.

Suppl. Fig 6

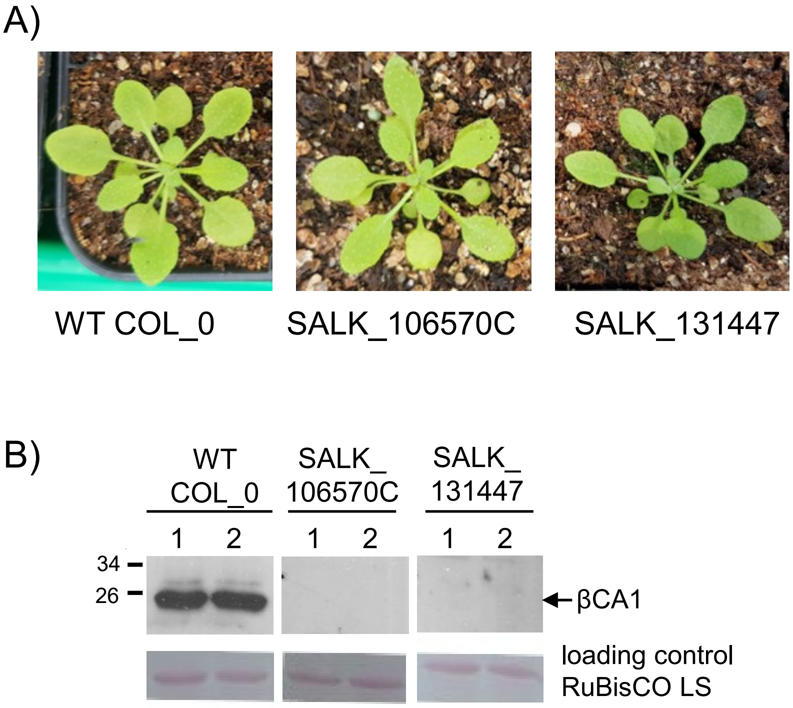

Figure S6 Characterization of *βca1* knock out lines (SALK_106570C and SALK_131447). A) Images of WT and *βca1* plants. B) Western Blot with βCA1 antibody of whole leaf protein extracts from WT and βCA1 knock out plants.

Table S 1 Primers used in this study for cloning.

Vector	Gene	AGI	Restriction enzyme		Primer Sequence 5'->3'
**pET15b**	TRX-f1	At3g02730	NdeI	Forward	AAAACATATGAGCTTAGAAACCGTTAATGTC
			BamHI	Reverse	TTTTGGATCCTCATCCGGAAGCAGCAGACCTC
**pET15b**	TRX-m1	At1g03680	NdeI	Forward	AAAACATATGGAAGCTCAGGACACTGCTACAG
			BamHI	Reverse	TTTTGGATCCTTACAAGAATTTGTTGATGCTG
**pET15b**	TRX-m2	At4g03520	NdeI	Forward	AAAACATATGGAAGCTCAGGAAACTACTAC
			BamHI	Reverse	TTTTGGATCCTCATGGCAAGAACTTGTC
**pET15b**	TRX-m4	At3g15360	NdeI	Forward	AAAACATATGGAGGCTCAGGACACCACTG
			BamHI	Reverse	TTTTGGATCCTTACTCGACCAAGAATCTTTCTATAG
**pET15b**	TRX-x	At1g50320	NdeI	Forward	AAAACATATGGGCGGAATCAAAGAGATTG
			BamHI	Reverse	TTTTGGATCCTTAAGCAACAGATATTGAGTTCAAG
**pET15b**	TRX-y1	At1g76760	NdeI	Forward	AAAACATATGGAAGCCAAGAAGCAGACATTTG
			BamHI	Reverse	TTTTGGATCCCTATGGCTTCACTTTTAGAGAATC
**pET15b**	TRX-y2	At1g43560	NdeI	Forward	AAAACATATGGCAGCAAAGAAGCAAACTTTC
			BamHI	Reverse	TTTTGGATCCCTACTGTTTCACTTGCAAAG
**pET15b**	CDSP32	At1g76080	NdeI	Forward	AAAACATATGGCTGGAGCGGCGTCTC
			BamHI	Reverse	TTTTGGATCCTAATAAGTGACACGAACGCCTG
**pEXP5 NTOPO**	TRX-f2	At5g16400	TOPO Cloning	Forward	TGTAGCTTAGAAACAGTGAATGTCACTGTTG
				Reverse	TCAGCCTGACCTTGCTGCTTC
**modified TOPO**	TRX-m3	At2g15570	BamHI	Forward	AAAAGGATCCCGCAGCTGAAGTTACACAAC
			AgeI	Reverse	TTTTACCGGTCATGAGTTCAAGACTCTTTCAATG
**pet28a**	NTRC	At2g41680	NdeI	Forward	AAAACATATGTCAGGAGGCGAGATTATCG
			BamHI	Reverse	TTTTGGATCCTTTATTGGCCTCAATGAATTCTC
**pET28a**	βCA1	At3g01500	BamHI	Forward	AAA AGG ATC CGC TCT TCA GAC AGG TAC TTC
			XhoI	Reverse	TTT TCT CGA GCT ACA GCT TCC AAT GTA GTA TGG
**pET28a**	βCA1 C167S	At3g01500	aqua cloning	Forward	TAAGTACATGGTGTTTGCTTCTTCAGACTCACGTGTGTGTCCATCA
				Reverse	AGAAGCAAACACCATGTACTTAGGACTTTGACCCTTTGCGAGCTCAC
**pET28a**	βCA1 C173S	At3g01500	aqua cloning	Forward	TCTCCATCACACGTTCTGGACTTTCAGCCAGGAGATGCCTTC
				Reverse	TCCAGAACGTGTGATGGAGACACACGTGAGTCTGAACAAG
**pET28a**	βCA1 C230S	At3g01500	aqua cloning	Forward	TGTGGTGATAGGACACAGTGCATCTGGTGGGATCAAAGGGCTTATGTC
				Reverse	AGATGCACTGTGTCCTATCACCACAATGTTCTCCACCTTAAGGTGTAAGAC
**pET28a**	βCA1 C257S	At3g01500	aqua cloning	Forward	TCAAAATCTCTTTACCAGCCAGGTCAAAGGTTATATCAG
				Reverse	GGCTGGTAAAGAGATTTTGACCCAGTCCTCTATGAAGTC
**pET28a**	βCA1 C277S	At3g01500	aqua cloning	Forward	AGATCAATCTGGCCGATGTGAAAGGGAGGCGGTGAATG
				Reverse	CACATCGGCCAGATTGATCTTCAAAGGCTGAATCTCCAAG
**pET28a**	βCA1 C280S	At3g01500	aqua cloning	Forward	GTGGCCGATCTGAAAGGGAGGCGGTGAATGTTTCACTAGC
				Reverse	CTCCCTTTCAGATCGGCCACATTGATCTTCAAAGGCTGAATCTC
**pet28a split primer**			aqua cloning	Forward	CGTGTAAGGGGGATTTCTGTTCATGGGGGTAATGATACCGATGAAACGAGAGAG
				Reverse	CCCCCATGAACAGAAATCCCCCTTACACGGAGGCATCAGTGACCAAACAG
